# Psych Socs: student-led psychiatry societies, an untapped resource for recruitment and reducing stigma

**DOI:** 10.1192/bjb.2019.88

**Published:** 2020-06

**Authors:** Haridha Pandian, Zahra Mohamedali, George E. Chapman, Patricia Vinchenzo, Suhana Ahmed, Zoé Mulliez, Helen Bruce, Wendy Burn, Ania Korszun, Derek K. Tracy

**Affiliations:** 1 Medical School, King's College London, UK; 2 College of Medical and Dental Sciences, University of Birmingham, UK; 3 Hampshire Hospitals NHS Foundation Trust, Hampshire, UK; 4 Queen's University Belfast, UK; 5 South West London & St Georges NHS Foundation Trust, UK; 6 Royal College of Psychiatrists, UK; 7 Great Ormond Street Institute of Child Health, University College London, UK; 8 Centre for Psychiatry, Wolfson Institute of Preventative Medicine, Queen Mary University of London, UK; 9 Oxleas NHS Foundation Trust, UK; 10 Department of Psychosis Studies, Institute of Psychiatry, Psychology & Neuroscience, King's College London, UK

**Keywords:** Psych Soc, psychiatry society, recruitment, stigma and discrimination

## Abstract

Medical recruitment and retention are national problems. Psychiatry has been more affected than many specialties, as a result of stigma from the public and other healthcare professionals. The Royal College of Psychiatrists has undertaken several initiatives to redress this, notably the ‘Choose Psychiatry’ campaign. In this editorial we argue that student-led university psychiatry societies are a wonderful but frequently untapped resource to help attract the brightest and best medical students to our profession. We describe the activities of three ‘Psych Socs’ across the UK and propose next steps to continue this work.

2019 saw the best psychiatry core trainee recruitment for some years, with 92% of places filled.^1^ However, the figures for higher training remain a concern, with a cumulative fill rate of 52% and considerable regional variation in both core and higher training.^1^ Staff recruitment and retention remain key challenges across the National Health Service,^2^ with overall vacancies predicted to double by 2030.^3^ Psychiatry has historically faced unique difficulties, not least stigmatising attitudes from the public, other doctors and medical students,^4^ and we need to remain active and focused on attracting the brightest and best to our profession. The time at medical school is a key period when attitudes and beliefs about psychiatry are most susceptible to change,^5^ and students' personal experience of psychiatry has been described as the ‘critical variable’ in recruitment rates.^6^

A survey by Curtis-Barton and Eagles^7^ reported ‘push’ factors that discouraged students from choosing psychiatry as a career, including a perceived lack of scientific evidence underpinning diagnoses, the perception that patients generally have a poor prognosis, the amount of ‘paperwork’ in the specialty and general stigma about mental illness. A qualitative study conducted at the University of Bristol echoed these findings, adding that the emotional burden of seeing patients at the lowest point of their lives and the focus on ‘non-medical’ social issues were also reasons reported by students choosing not to pursue psychiatry.^8^

Psychiatry and general practice have been shown to attract most negative comments or ‘bashing’ from academic staff, doctors and students.^9^ Halder *et al*^10^ found that across 18 medical schools, 16% of medical students considered psychiatry as a future career upon entering medical school; by the final year 17% reported still seriously considering it, but only 3% had decided to actually pursue the specialty. A total of 27% of students reported that they had changed their future specialty choice as a result of ‘direct bashing’.^9^ By the latter years of undergraduate study, the point at which many students begin direct psychiatry teaching, many more medical students held negative beliefs about the specialty less adaptable to change.^11,12^

Conversely, a positive experience of a psychiatry rotation and extracurricular enrichment ‘pull factor’ opportunities, such as engaging electives and special study modules, can ignite an interest and have been found to be one of the strongest predictors of career choice.^5,10^

## Royal College strategies

The Royal College of Psychiatrists (RCPsych) has attempted to tackle both push and pull factors. In 2016, it ran the ‘Anti-BASH’ campaign,^12^ utilising the Twitter hashtag ‘#banthebash’ to identify and address badmouthing, attitudes and stigmatising in healthcare, in particular from clinicians in other specialties.

More recently, the College has emphasised positive pull factors, offering free Student Associate membership^13^ to the College and providing free access to network events and College journals.^14^ The ‘Choose Psychiatry’ campaign^15^ has adopted a strategy of demonstrating the many positive aspects of a career in psychiatry, with the use of short video-clips encompassing real-life patient stories and the impact that psychiatry has made on their lives, as well as pieces by psychiatrists explaining why they chose and enjoy their careers. This encourages viewers to join the conversation on social media, using the hashtag #choosepsychiatry.

The 1-year ‘Psych Star’ scheme supports medical students through mentoring and financial support.^16^ A 2-year ‘Foundation Fellowship’ offers a parallel route for foundation year doctors, with both schemes supporting candidates to act as local ambassadors for promotion of the specialty. Although it is not possible to causally link these efforts and the recent improved recruitment, we should still acknowledge the College's work to achieve this result.

## Psychiatry societies

A less-explored area is the momentum generated by university psychiatry societies (Psych Socs), which are led by students with support from clinicians and the RCPsych. These bottom-up initiatives host diverse local events with the aims of raising the profile of mental health issues, challenging stigmatising attitudes by increasing understanding of the central role that psychiatry has in medicine and inspiring students to choose psychiatric careers. They also come together annually for a national Psych Soc conference, hosted by one of the organisations. Here we describe the types of activities undertaken across three UK societies, in London, Birmingham and Belfast.

### Augmenting the syllabus: guest lectures and exam practice

The societies host diverse free guest lectures across the range of psychiatric subspecialties complementing and extending the undergraduate curriculum. Mukherjee *et al*^5^ argued that placing a particular focus on liaison psychiatry during undergraduate teaching allows students to appreciate that psychiatry has a central role in the aetiology and outcome of many medical disorders. In our Psych Socs, we have had particularly positive experiences when collaborating with other university societies to promote this understanding (see Box 1 for more detail): for example, gastroenterology societies to discuss eating disorders; paediatrics and obstetrics and gynaecology societies to discuss perinatal psychiatry, autism and neurodevelopmental disorders; emergency medicine societies to learn about patients presenting in crisis; and oncology societies to discuss psycho-oncology. Some Psych Socs also organise additional examination practice, for example via mock OSCEs and history-taking workshops, as well as providing more links and discussion around psychiatry electives and research opportunities.

Box 1 Examples of well-received Psych Soc events

- ‘Evolution and the brain’, discussing how brain functioning and psychopathology can be understood using evolutionary perspectives.
- ‘Real people sharing real stories’, five students shared their personal experiences of living with mental illness.
- ‘Time to put the psychedelics back into psychiatry?’, a discussion on psychedelics in modern psychiatry.
- ‘Trauma and violence’ with trauma surgeons, a psychiatrist and young victims of knife crime discussing post-traumatic stress disorder.
- ‘Through the lens’ mental health photography workshop with the Health and Humanities society, discussing the portrayal of borderline personality disorder in the arts.
- Psychiatric themes in Don Quixote and Othello syndrome in ‘A Winter's Tale’
- ‘Homelessness and healthcare’ with individuals who had been street homeless, describing how this impacted their ability to access care, and their experiences of living in the streets.
- ‘Disfigurement and quality of life’, with maxillo-facial surgeons and psychiatrists discussing the impact of facial surgery on perceived quality of life.
- ‘Mental health in developing countries’ hosted by psychiatry trainees and ‘Students for Global Health’, discussing different practices in other countries, and career opportunities in international assistance.
- ‘Not guilty by reason of insanity’, exploring the roles of forensic psychiatrists.
- ‘Mental disorder and autonomy: classical and romantic perspectives’, a seminar co-hosted with a Philosophy Society discussing varying philosophical views of mental illness across time.
- ‘Sex and psychiatry’ seminar with the university ‘Sexpression’ group, discussing psychiatric bases for dyspareunia, tocophobia and so forth.

Talks on novel fields not typically covered in lectures are usually very popular, such as evolutionary psychiatry, psychosexual medicine and cutting-edge research (for example, therapeutic use of psychedelics). These have the additional value of attracting a wider range of medical students who might not attend more ‘standard’ psychiatry talks, and indeed are often enriched by pulling in students from different disciplines, such as philosophy and the arts, and members of the local community. This reinforces a message of mental health at the centre of medicine and society, and challenges stigmatising attitudes.

Crucially, as membership is open to all students, these events are great opportunities to attract pre-clinical medical students several years before their psychiatry teaching and placements, and potentially before more significant exposure to any ‘psychiatry bashing’.

### Tackling stigma and discussing student mental health

Brown and Ryland^17^ emphasised the importance of involving people with mental health disorders in student education, particularly those who have recovered, as placements are often too short for students to experience this. Psych Soc speakers are encouraged to explore relevant case studies, and we endeavour to invite speakers with lived experience. One Psych Soc has published a single-arm pre–post comparison study, which demonstrated statistically significant reductions in student stigma in the domains of knowledge, attitude and behaviour following exposure to a perinatal event when a mother spoke of her personal journey.^18^

Students can feel less able to disclose their own mental health problems because of perceptions of peers’ negative views,^12^ and successful Psych Soc events have also discussed and promoted resources for student well-being especially during examination periods. Psychiatrists have helped with this, with events on ‘Mental Health in Healthcare’ and ‘Bipolar Disorder: Don't Believe Everything You Hear’ hosted by health care professionals who themselves have recovered from psychological problems.^19^ This also addressed psychological challenges and pressures students might face once qualified.

### Work in the arts

Broader Psych Soc initiatives involving the arts have proved very popular. These have included a student film and book club (one in conjunction with the local psychiatry trainees’ book group) and exploring the perception of mental illness in popular literature and media. Popular talks have discussed the portrayal of psychopathology in historic literature, such as Othello syndrome in ‘A Winter's Tale’ and wider psychiatric themes in ‘Don Quixote’. The ‘MedFest’ film festival is a popular international event for Psych Socs and mental health more broadly, displaying and discussing short films that touch on pertinent issues in mental health.

### Dissemination through new tools: social media

Psych Socs successfully use a range of social media, from Facebook to Twitter and Instagram, and more ‘old-fashioned’ email to reach students. These regularly share information regarding wider opportunities, such as summer schools (unlike many parallel schemes in other specialties, most of these are free), RCPsych events, prizes and bursaries, student-selected components in psychiatry, research and elective opportunities and so forth. They also provide guidance and encouragement to students on becoming College Associate Members of the College, and advertise College resources, articles and podcasts. Anecdotally, many students have informed us that Psych Soc posts on social media have alerted them to opportunities of which they had previously been unaware.

In October 2019, Queen's University Belfast ‘Mind Matters’ Psych Soc hosted a highly successful 1-hour ‘Twitter Takeover’. Numerous psychiatrists and other Psych Socs across the country participated, answering questions on how medical students can get involved with psychiatry early, personal reasons for choosing psychiatry, upcoming events and interesting books and articles relevant to students. Twitter in particular affords an opportunity to engage and connect with the many psychiatrists and medical students online, unhindered by distance.

### Starting a Psych Soc

Medical students and psychiatrists interested in starting a Psych Soc at their own local university should firstly endeavour to recruit a core committee of students for the academic year. The committee should attempt to make contact with the Undergraduate Lead for Psychiatry at their university, the RCPsych regional division and other local psychiatrists. Such contacts may be called upon to act as speakers at evening lecture events, mock OSCE examiners and mentors.

Psych Socs should also contact the RCPsych to receive funding for events, as each university society receives a grant of £500 per annum. The College also offer free promotional material such as pens, key rings and leaflets, which can be handed out as ‘freebies’ during events. The RCPsych website includes detailed advice for setting up a local Psych Soc, event ideas and contact details for useful stakeholders.^20^

## Psychiatrists’ perspectives and next steps

As senior clinicians, we recall the difference that enthusiastic and passionate trainers, teams and rotations made to our career choices at all stages, from medical school through to our own training.^21^ Sadly, we have also all experienced the negative effect of ‘bashing’ of psychiatry and our patients by other medical students and doctors. All psychiatrists need to remain proud advocates for our profession and remember that every contact counts. The recent College initiatives for recruitment appear to be paying dividends with the positive message of ‘Choose Psychiatry’ particularly pleasing.

The Psych Socs, however, speak to students in a way we cannot, and it is heartening to see the positive energy they generate. Enthusiastic medical students deliver the compelling message that psychiatry is a mainstream part of medicine and offers a diverse and rewarding career and a flexible work–life balance. Their bottom-up initiatives relevant to their local teaching and training, identification of gaps and novel areas they wish to explore, and the fun, interesting and culturally broader events in turn have refreshed us. The Psych Socs typically offer compensation to speakers through covering their expenses, but in our experience the real payment is the pleasure of sharing and contributing to their enthusiasm.

Several next steps can be recommended both locally and nationally. Students require enthusiastic engagement from local psychiatrists: as guest speakers, mock OSCE examiners and mentoring via ‘buddy schemes’. The relationship should be reciprocal: assisting students with areas they request as needing redressing, but also using our contacts and experience to suggest and link-up additional input. Students often need discrete guidance in organising events and making sure that these are well balanced in the views that are expressed.

Nationally, the RCPsych has created a supportive linking webpage to share ideas and learning; this and the annual National Student Psychiatry Conference need to be nurtured and grown. In a time of austerity, there are inevitable challenges about ‘who funds’ travel and attendance, but medical schools and the College need to continue to encourage and maximise subsidised student engagement, including through poster presentations, oral presentations, student sections and prizes. This is not just a ‘central’ issue, it falls to all divisions and faculties to review their engagement. We propose that Psych Socs are an excellent opportunity for outreach to catch the best future colleagues. As a College we need to be better at recognising, celebrating and sharing what is working with our medical students. A recently published RCPsych report^22^ makes explicit recommendations for a range of initiatives on enhancing interest in psychiatry, including developing medical student psychotherapy schemes and Balint groups, and better working with Psych Socs. The College's Choose Psychiatry Committee has an initiative to make sure that each Psych Soc for the next academic year has a link senior member of the Committee to help support local initiatives.

We believe that university Psych Socs are a secret, but as yet not fully exploited, tool to improve recruitment into psychiatry, as well as promoting respect for the profession and mental health amongst those who do not become psychiatrists. They offer a valuable opportunity for students and psychiatrists to work together, and for us to continue to encourage the brightest and best to join what we know to be the most rewarding of medical specialties.

## About the authors

**Haridha Pandian** is a medical student at the Medical School of King's College London, UK, and President of the King's College London Psych Soc. **Zahra Mohamedali** is a medical student at the Medical School of King's College London, UK, and Vice President of the King's College London Psych Soc. **George E. Chapman** is the former Vice President of the Psych Soc in the College of Medical and Dental Sciences, University of Birmingham, and a foundation year doctor at Hampshire Hospitals NHS Foundation Trust, Hampshire, UK. **Patricia Vinchenzo** is a medical student at Queen's University Belfast, UK, and President of Queen's University 'Mind Matters' Psych Soc. **Suhana Ahmed** is a consultant psychiatrist at South West London & St Georges NHS Foundation Trust, UK. **Zoé Mulliez** is Policy and Campaigns Manager at the Royal College of Psychiatrists, UK. **Helen Bruce** is a Consultant Psychiatrist at Great Ormond Street Institute of Child Health, University College London, UK. **Wendy Burn** is President at the Royal College of Psychiatrists, UK. **Ania Korszun** is Professor of Psychiatry and Education in the Wolfson Institute of Preventative Medicine, Queen Mary University of London, UK. **Derek K. Tracy** is a consultant psychiatrist and clinical director at Oxleas NHS Foundation Trust and a senior lecturer in the Department of Psychosis Studies, Institute of Psychiatry, Psychology & Neuroscience, King's College London, UK.
